# Whole-genome sequencing provides insights into a novel species: *Providencia hangzhouensis* associated with urinary tract infections

**DOI:** 10.1128/spectrum.01227-23

**Published:** 2023-09-21

**Authors:** Xu Dong, Yuyun Yu, Jiaying Liu, Dan Cao, Yanghui Xiang, Kefan Bi, Xin Yuan, Shengchao Li, Tiantian Wu, Ying Zhang

**Affiliations:** 1 State Key Laboratory for Diagnosis and Treatment of Infectious Diseases, National Clinical Research Center for Infectious Diseases, Collaborative Innovation Center for Diagnosis and Treatment of Infectious Diseases, The First Affiliated Hospital, Zhejiang University School of Medicine, Hangzhou, China; 2 Department of Laboratory Medicine, The First Affiliated Hospital, Zhejiang University School of Medicine, Hangzhou, China; University of Texas Southwestern Medical Center, Dallas, Texas, USA

**Keywords:** novel species, *Providencia hangzhouensis*, *Providencia rettgeri*, *Providencia huaxiensis*, carbapenem resistance, pan-genome analysis

## Abstract

*Providencia rettgeri* is a clinically significant opportunistic pathogen that is involved in urinary tract infections. Due to the resolution limitations of identification, distinguishing *P. rettgeri* from closely related species is challenging by commercial biochemical test systems. Here, we first reported a novel species, *Providencia hangzhouensis*, which had been misidentified as *P. rettgeri*. Exhibiting ≤91.97% average nucleotide identity (ANI) and ≤46.10% in silico DNA-DNA hybridization values with all known *Providencia* species, *P. hangzhouensis* falls well beneath the established species-defining thresholds. We conducted a population genomics analysis of *P. hangzhouensis* isolates worldwide. Our study revealed that *P. hangzhouensis* has emerged in many countries and has formed several transmission clusters. We found that *P. hangzhouensis* shared the highest ANI values (91.54% and 91.97%) with *P. rettgeri* and *P. huaxiensis*, respectively. The pan-genome analysis revealed that these three species possessed a similar component of pan-genomes. Two genes associated with metabolism, *folE2* and *ccmM*, were identified to be specific to *P. hangzhouensis*. Furthermore, we also observed that carbapenem-resistance genes frequently occur in *P. hangzhouensis* with the *bla*_IMP-27_ being the most prevalent (46.15%; 36/78). The emergence of *P. hangzhouensis* is often accompanied by extended-spectrum β-lactamase and carbapenem-resistance genes, and calls for tailored surveillance of this species as a clinically relevant species in the future.

**IMPORTANCE:**

Our study has identified and characterized a novel species, *Providencia hangzhouensis*, which is associated with urinary tract infections and was previously misidentified as *Providencia rettgeri*. Through this study, we have identified specific genes unique to *P. hangzhouensis*, which could serve as marker genes for rapid PCR identification. Additionally, our findings suggest that the emergence of *P. hangzhouensis* is often accompanied by extended-spectrum β-lactamase and carbapenem-resistance genes, emphasizing the need for attention to clinical management and the importance of accurate species identification and proper drug use.

## INTRODUCTION

The *Providencia* genus is a member of the *Enterobacterales* order, which constitutes the tribe *Proteeae* along with the *Proteus* and *Morganella* genera. *Providencia* includes Gram-negative bacteria that produce urease and are accountable for causing diverse human infections (1). Among the species belonging to *Providencia*, *P. rettgeri* and *P. stuartii* are the most frequent etiologic agents of catheter-associated urinary tract infections, particularly among elderly patients with long-term indwelling urinary catheters (1). Moreover, there are several case reports of other infections associated with *P. rettgeri*, such as ocular diseases (2), peritonitis (3), and neonatal septicemia (4), indicating that this pathogen has the potential to cause serious infections beyond catheter-associated urinary tract infections.

Precise species and subspecies classification of bacterial isolates is fundamental for understanding their epidemiology, pathogenesis, and microbiological characteristics. The approaches for prokaryotic species delineation include DNA-DNA hybridization (DDH), 16S rRNA identification, and average nucleotide identity (ANI) analysis. Although DDH analysis holds the distinction of being the gold standard for species classification (5), it is known to be error-prone, with low reproducibility and time-consuming procedures. Furthermore, it is widely acknowledged that analyzing the 16S rRNA gene sequence alone is insufficient for accurate bacterial species assignment (6). As a result, with the significant reduction in the cost of sequencing, genome-based ANI and *in silico* digital DNA-DNA hybridization (dDDH) analyses are becoming increasingly popular in microbiology laboratories (7). Pairwise ANI analysis with a cutoff of ≥96% and dDDH with a cutoff of ≥70.0% have been widely used for precise species identification (8 – 11).

In this study, we performed whole-genome sequencing of a clinical isolate of *Providencia rettgeri*, PR-310, and found it to represent a novel species with 92.03% identity with *P. rettgeri*, which we name *Providencia hangzhouensis*, through ANI analysis. We then examined the isolates labeled as *P. rettgeri* in the NCBI database and discovered that the novel species had been mistakenly identified as *P. rettgeri* previously. Additionally, we investigated the population structure and genetic diversity of *P. hangzhouensis* using comparative genomic and population genomic analysis.

## RESULTS

### *Providencia hangzhouensis* represents a novel species distinct from *Providencia rettgeri*

Since strain PR-310, which was isolated from a patient with urinary tract infection and identified as *Providencia rettgeri* by Matrix-Assisted Laser Desorption/Ionization - Time of Flight mass spectrometry (MALDI-TOF MS) (Vitek MS system, bioMerieux, France), is an uncommon clinical opportunistic pathogen and displayed resistance to cephalosporins; this strain was then subjected to whole-genome sequencing. The complete genome of PR-310 is composed of a chromosome and two plasmids, named pPR-310_79 and pPR-310_2. The chromosome of PR-310 spans a length of 4,515,965 bp and exhibits a Guanine-Cytosine (GC) content of 40.26%. Additionally, the lengths of the two plasmids are 79,375 and 2,683 bp, respectively. Analysis of the 16S rRNA gene sequences, taken from the EzBioCloud database (12) and corroborated by a derived phylogenetic tree (Fig. S1A), confirmed that the PR-310 strain classifies within the *Providencia* genus. This strain exhibited the highest sequence identity of the 16S rRNA gene with *P. rettgeri* DSM 4542 (99.73%; AM040492) and *P. huaxiensis* WCHPr000369 (99.66%; CP031123). Nevertheless, the limitations of using the 16S rRNA sequence for taxonomic assignment are widely acknowledged (13). Hence, we performed a whole-genome-based phylogenetic analysis on PR-310 and type strains within the *Providencia* genus. Our results affirmed that PR-310 indeed belongs to the *Providencia* genus, and shares the closest relationship with *P. rettgeri* NCTC11801 (Fig. S1B). A comparative analysis of the ANI (77.20%–91.97%) and dDDH (21.20%–46.10%) values of strain PR-310 with other type strains (Table 1) revealed these to be significantly below the accepted thresholds for species classification (ANI > 96; dDDH > 70%). Thus, the collective results suggest that strain PR-310 should be regarded as a novel species, which we propose to name *Providencia hangzhouensis*, rather than *P. rettgeri*.

**TABLE 1:** Average nucleotide identity and *in silico* DNA-DNA hybridization values between strain PR-310 and the type strains of *Providencia* species

Species and strain	Assembly accession	ANI (%)	dDDH (%)
*P. alcalifaciens* ATCC 51902	CP023536.1	77.76	21.20
*P. manganoxydans* LLDRA6	CP067099.1	77.25	21.70
*P. rustigianii* NCTC12026	NZ_UGUA01000002.1	77.99	21.30
*P. burhodogranariea* DSM 19968	NZ_KB233222.1	77.15	21.50
*P. heimbachae* NCTC12003	LS483422.1	79.16	22.20
*P. sneebia* DSM 19967	CM001773.1	77.08	21.70
*P. huaxiensis* WCHPr000369	CP031123.2	91.97	46.10
*P. rettgeri* NCTC11801	GCA_900455085.1	91.54	45.40
*P. stuartii* ATCC 25827	GCA_000154865.1	77.20	21.50
*P. thailandensis* KCTC 23281	NZ_BMYH01000001.1	77.25	21.30
*P. vermicola* DSM 17385	NZ_CP048796.1	81.49	23.90

To delve deeper into this novel species, represented by strain PR-310, we included in our study the genome sequences of *P. rettgeri* and *P. huaxiensis*, obtained from NCBI database. These species, with an ANI value>90%, provide a relevant genomic context for a more comprehensive analysis due to their close genetic relationship with *P. hangzhouensis*. Interestingly, of the 336 *P*. *rettgeri* sequences retrieved, 201 were classified as *Providencia* genus, and 11 belonged to *P. huaxiensis*. Only 52 isolates were identified as true *P. rettgeri* through ANI analysis. Additionally, we identified 99 isolates with ANI values >96% to PR-310, indicating that *P. hangzhouensis* had appeared previously but had been misidentified as *P. rettgeri*.

### General characteristics of the *P. hangzhouensis* isolates

Biochemical characteristics of strain PR-310 and type strains of other *Providencia* species are shown in Table 2. The strain PR-310 is negative for oxidase activity and is Gram-negative, motile, facultatively anaerobic, and rod-shaped. Colonies appear circular, raised, yellow, opaque, and smooth after 24 h of incubation at 37°C on nutrient agar. The strain is able to utilize acid from D-glucose, D-mannitol, inositol, L-rhamnose, and amygdalin, but not from D-sorbitol, sucrose or melibiose, and D-arabinose. It tests positive for deaminase activity but negative for β-galactosidase, arginine dihydrolase, ornithine decarboxylase, lysine decarboxylase, and gelatinase. Additionally, the strain is positive for urease activity, indole production, and Voges-Proskauer reaction, and can utilize citrate, but does not produce H_2_S. The strain PR-310 can be distinguished from all other *Providencia* species by its positive response to acetoin production and citrate utilization tests, while it is unable to metabolize melibiose.

**TABLE 2:** Biochemical characteristics of strains PR-310 and type strains of other *Providencia* species^*a*^

Characteristic	PR-310	1	2	3	4	5	6	7	8	9	10	11
β-Galactosidase	−	−	−	−	−	−	−	−	−	−	+	−
Arginine dihydrolase	−	−	−	−	−	−	−	−	−	−	+	−
Lysine decarboxylase	−	−	−	−	−	−	−	−	−	−	−	−
Ornithine decarboxylase	−	−	−	−	−	−	−	−	−	−	+	−
Citrate utilization	+	+	−	−	+	+	−	−	−	+	+	−
H_2_S production	−	−	−	−	−	−	−	−	−	−	−	−
Urea hydrolysis	+	+	−	−	−	+	+	−	+	−	−	−
Deaminase	+	+	+	+	+	+	+	+	+	+	+	+
Indole production	+	+	+	+	−	+	+	+	+	+	−	+
Acetoin production	+	−	−	−	−	−	+	−	−	−	+	−
Gelatinase	−	−	−	−	−	−	−	−	−	−	+	−
D-glucose	+	+	+	+	+	+	+	+	+	+	+	+
D-mannitol	+	+	−	+	−	+	−	−	+	−	+	+
Inositol	+	+	−	+	−	+	+	−	−	+	+	+
D-sorbitol	−	−	−	−	−	−	−	−	+	−	+	−
L-rhamnose	+	−	−	−	−	−	+	−	−	−	+	−
Sucrose	−	+	−	−	−	−	−	−	−	+	+	−
Melibiose	−	−	−	−	−	−	−	−	−	−	+	−
Amygdalin	+	−	−	−	−	+	+	−	+	−	−	−
Arabinose	−	−	−	+	−	−	−	−	−	−	+	+

^*a*^ Strains: 1, *P. manganoxydans* LLDRA6; 2, *P. alcalifaciens* DSM 30120; 3, *P. burhodogranariea* DSM 19968; 4, *P. heimbachae* DSM 3591; 5, *P. huaxiensis* KCTC 62577; 6, *P. rettgeri* DSM4542; 7, *P. rustigianii* DSM 4541; 8, *P. sneebia* DSM 19967; 9, *P. stuartii* DSM 4539; 10, *P. thailandensis* KCTC 23281; 11, *P. vermicola* DSM 17385. Data for species other than *P. hangzhouensis* PR-310 are from reference (14). + represents 90 to 100% positive reaction and − represents 0 to 10% positive reaction.

**Fig 1:**
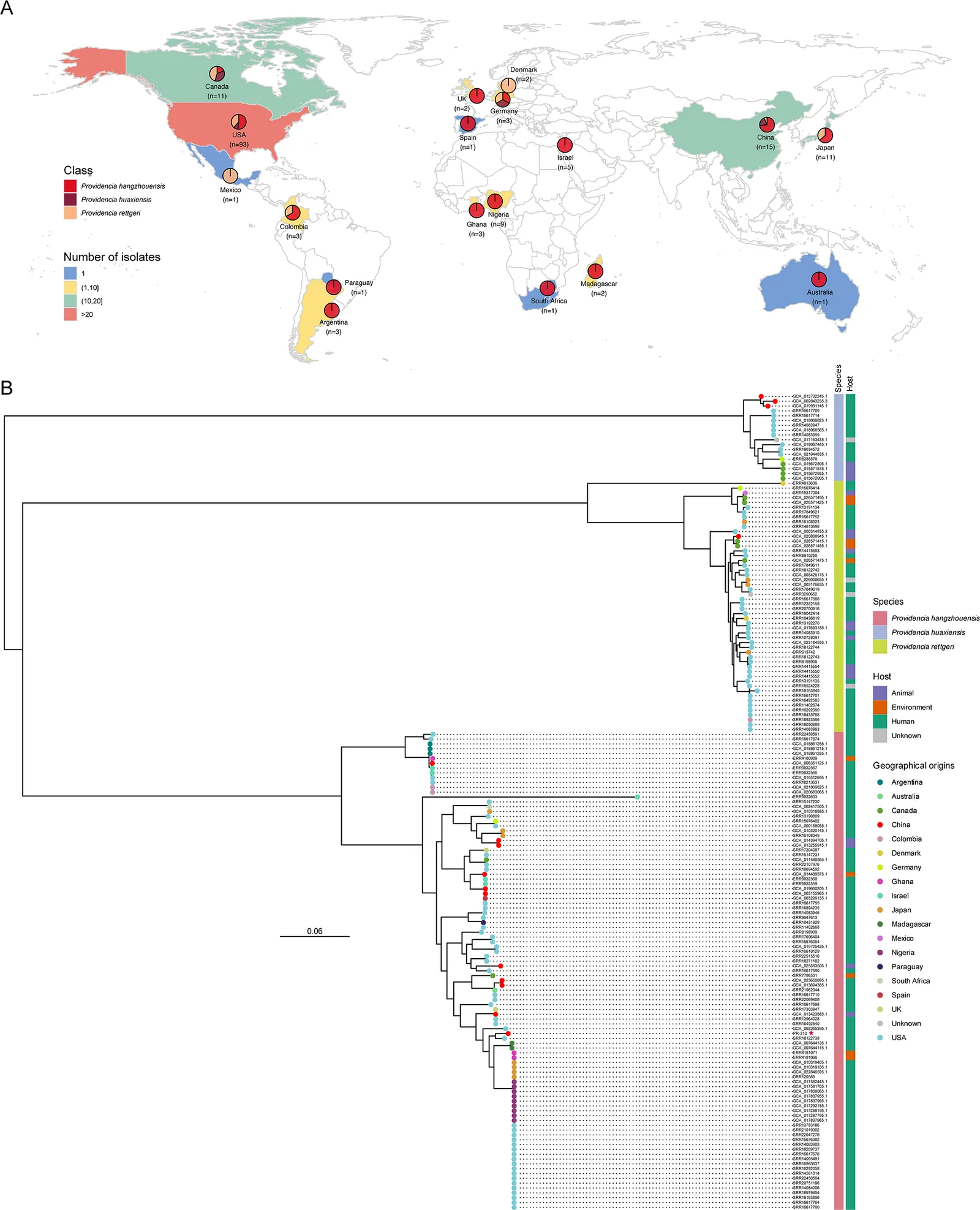
Global diversity of the *P. hangzhouensis*. (A) Geographical distribution of the 169 isolates in the study. (B) Core-genome-based maximum-likelihood phylogeny of the 169 isolates. The hues of the terminal nodes are indicative of the geographic source of each individual isolate.

Following quality control, we amassed a total of 169 genomes, composed of 99 from *P. hangzhouensis*, our primary focus and the species with dominance in both quantity and geographical distribution, as well as 52 and 18 from *P. rettgeri* and *P. huaxiensis*, respectively. These latter species were included due to their high ANI values >90, indicating a close genetic relationship with *P. hangzhouensis*. These genomes span 18 countries, with *P. hangzhouensis* being most prevalent in the United States (*n* = 48), followed by China (*n* = 11), and Nigeria (*n* = 9) (Fig. 1A). The average genome size of *P. hangzhouensis* is 4.69 Mb, with a range of 4.34–5.25 Mb and an average GC content of 40.40%. To identify core genes in the three species, we employed a 90% amino acid identity threshold. A total of 2,170 genes, covering a length of 2.08 Mb, were concatenated to generate a single core-genome alignment for the construction of the maximum-likelihood (ML) phylogeny. The 169 genomes were divided into three main branches in the phylogenetic tree according to their species correlation, which is consistent with the results of ANI and dDDH (Fig. 1B). Furthermore, *P. hangzhouensis* has the highest clinical isolation rate (90.91%), which is much higher than 68.38% of *P. rettgeri* and 72.22% of *P. huaxiensis*, indicating that *P. hangzhouensis* might be more likely to appear in the clinical settings.

The 99 *P*. *hangzhouensis* genomes were further divided into five main clusters based on 37,561 core non-recombinant single nucleotide polymorphisms (SNPs) (Fig. S2). To determine the potential transmission pairs among *P. hangzhouensis* isolates, we analyzed the number of SNPs that differentiate these isolates. As shown in Fig. S3, there were large variations in the number of SNPs among the strains, ranging from 0 to 24,075. However, we identified a large transmission cluster comprising 45 isolates spanning four countries (the United States, Japan, Ghana, and Nigeria) with SNPs ≤ 58, indicating high relatedness among these clones.

### Pan-genome analysis within species

**Fig 2:**
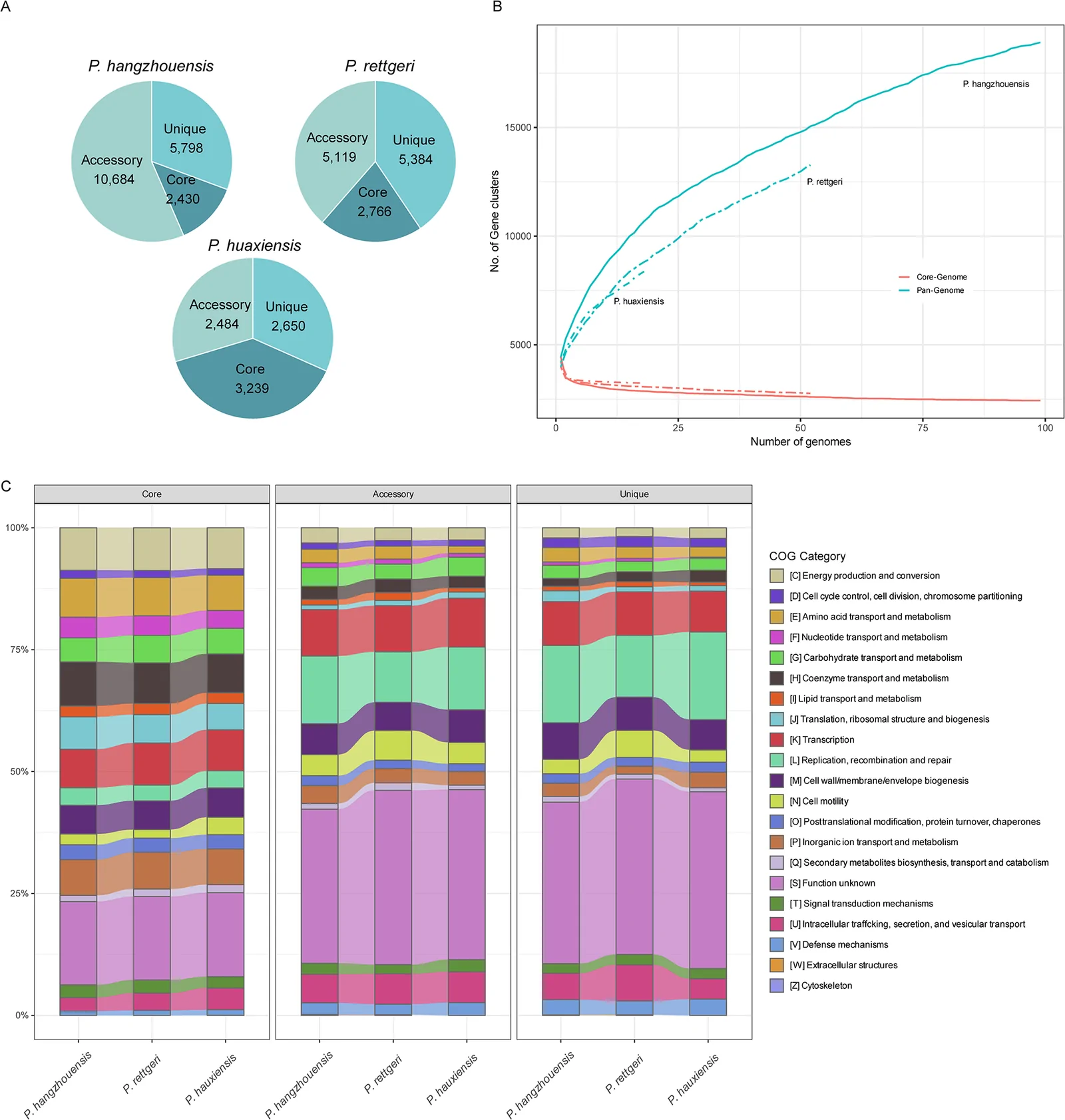
Pan-genome analysis and cluster of orthologous groups (COG) functional annotation of *P. hangzhouensis*, *P. rettgeri*, and *P. huaxiensis*. (A) Pie charts showing the distribution of core, accessory, and unique genes of three species. (B) Accumulation curves for pan-genome (blue) and core genomes (red). (C) Distribution of COG functional categories for core, accessory, and unique genomes.

To explore the genetic composition of individual species and the differences between species, we investigated the pan-genome features within three species. As shown in Fig. 2A, the pan-genome of *P. hangzhouensis* is composed of 18,912 gene families, of which 2,430 genes were core genes, 10,684 were accessory genes (genes present in two or more genomes), and 5,798 unique genes (a gene specific to a single genome). Similarly, there were 2,766 core genes, 5,119 accessory genes, and 5,384 unique genes in the *P. rettgeri* as well as 3,239 core genes, 2,484 accessory genes, and 2,650 unique genes in *P. huaxiensis*.

Analyzing the size of the pan-genome and its expansion or contraction upon the addition of new isolates can serve as a means for predicting the future rate of novel gene discovery within a given species (15, 16). As depicted in Fig. 2B, it is obvious that with the addition of genomes, the pan-genome sizes of the three species are increasing rapidly, while the core-genome size decreases and tends to be a plateau. In addition, we calculated the decay parameter *α*, where a value of *α* < 1.0 indicates that the size of the pan-genome is increasing and not limited by the number of genomes considered (15, 16). We obtained *α* values of 0.66, 0.67, and 0.75 by reusing 100 permutations for *P. hangzhouensis*, *P. rettgeri*, and *P. huaxiensis*, respectively. The results suggested that the pan-genomes of the three species are open, with a greater likelihood of discovering new genes as more genomes are sequenced in the future.

To gain a thorough understanding of the functional traits of each component in the pan-genome, we conducted a cluster of orthologous groups (COG) functional classification for core genes, accessory genes, and unique genes. The results revealed that the three species shared a similar pan-genomic composition in general (Fig. 2C). Specifically, both the accessory genes and unique genes were mainly abundant in replication, recombination, and repair, indicating that recombination was important for evolution.

### Genes potentially contributing to the success of *P. hangzhouensis*

**Fig 3:**
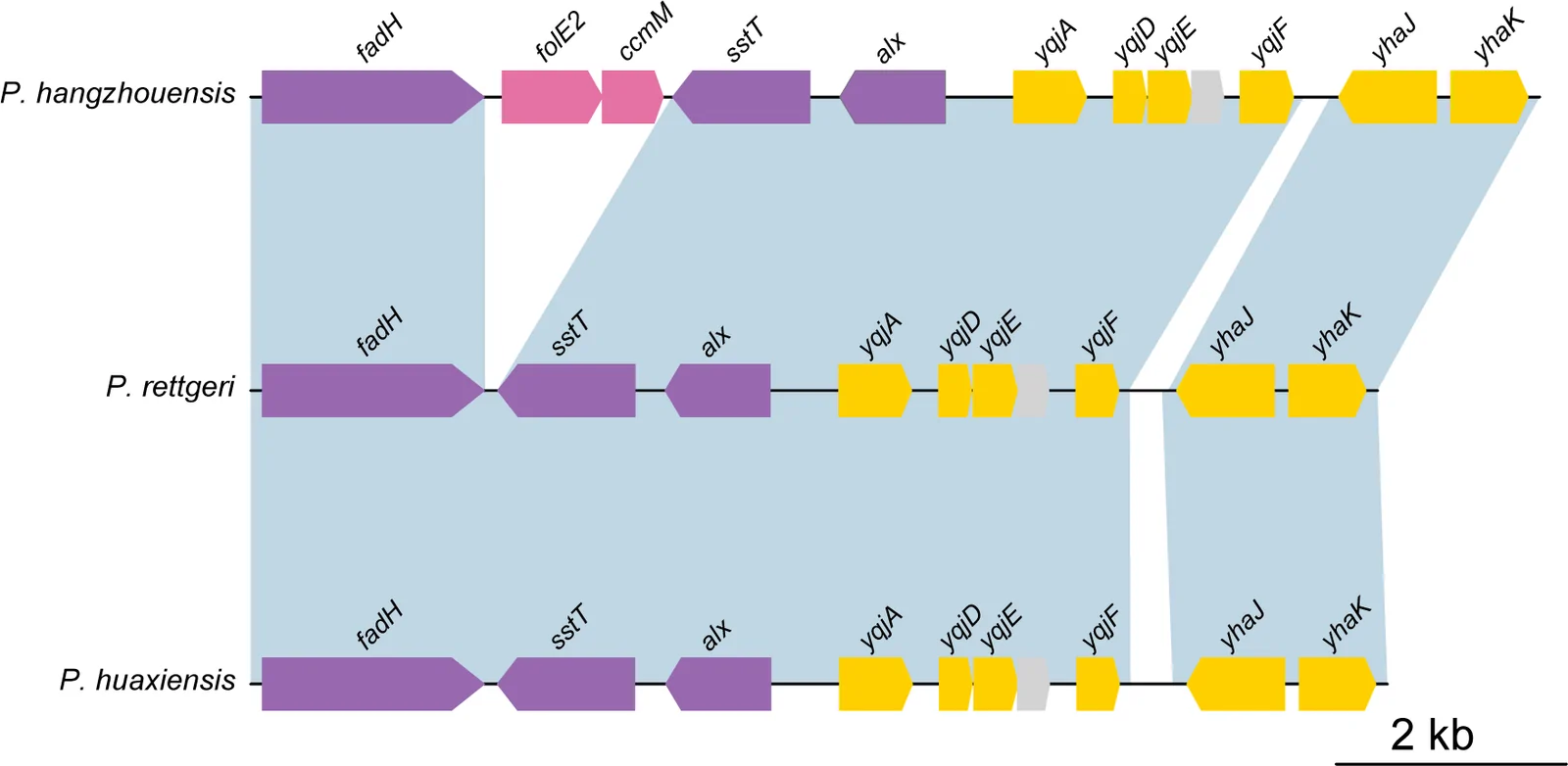
Comparative genetic environment upstream and downstream of *folE2* and *ccmM* among three species. Genes are shown as arrows and colored based on gene function classification. Those genes without direct gene names are marked with gray.

Combined with the occupancy of the database, the clinical isolation rate, and the only strain of *P. hangzhouensis* we have received in the past year, this suggests that *P. hangzhouensis* has evolved more successfully and might have higher fitness under hospital settings than the other two species. Only four genes specific to *P. hangzhouensis* were identified, two of which were non-adjacent hypothetical proteins and the remaining two adjacent genes, *folE2* and *ccmM*, were associated with the metabolic adaptation (Table S1). Comparative genomic analysis revealed that these two consecutive genes, *folE2* and *ccmM*, are located in a relatively conserved region of *P. hangzhouensis*, while they are absent in the other two species (Fig. 3). The presence of these two genes related to glucose metabolism might promote energy metabolism and contribute to their expansion and survival in certain settings. Notably, a BLAST search revealed that the *folE2*-like and *ccmM*-like genes are not present in the other *Providencia* species, nor in other genera. We next conducted an amino acid-level search and found that the *folE2* and *ccmM* proteins exhibited some similarities to those in other genera, particularly *Obesumbacterium proteus*, with the highest identity of 61.74% and 82.8% and coverage of 96% and 100%, respectively.

### Recombination in *P. hangzhouensis*

**Fig 4:**
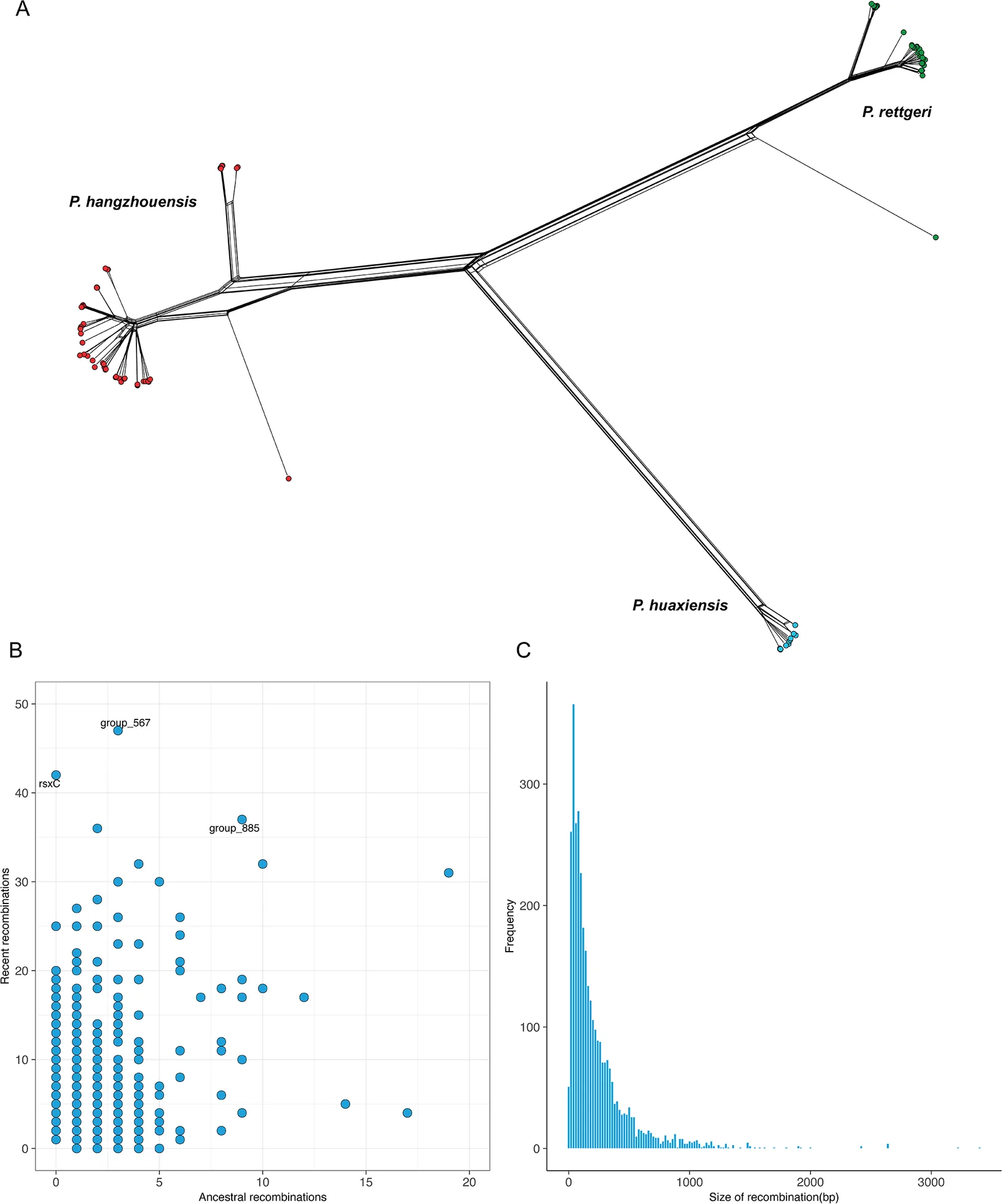
Genomic recombination of the *P. hangzhouensis*. (A) Phylogenetic network of the *P. hangzhouensis*, *P. rettgeri*, and *P. huaxiensis* based on the core-genome alignment. (B) Number of recent and ancestral recombination events in *P. hangzhouensis*. (C) Frequency histogram of the size of recombination events in the pan-genome of *P. hangzhouensis*.

To investigate the effect of recombination on *P. hangzhouensis*, we first analyzed the recombination events in three closely related species. We obtained a complex evolutionary network based on the core-genome alignment of three species (Fig. 4A), indicating that recombination has occurred over a considerable part of the genome. We next used fastGEAR to explore the recombined events among species and the results showed that there were few ancestral recombination (occurring during the speciation) and extensive recent recombination (occurring after the speciation) between species, contributing to the higher diversity of *P. hangzhouensis* genomes (Fig. S4).

To further investigate the recombination hotspot genes in *P. hangzhouensis* genomes, we performed analysis of each core gene and accessory gene alignment using the fastGEAR algorithm. A total of 2,840 genes were found to have experienced genetic recombination, accounting for 15.01% of the pan-genome, among which two gene families without specific gene name (group 567 and group 885) and a gene named *rsxC* showed the highest recombination frequency (Fig. 4B; Table S2). Group 567 represents an insulinase family protein related to proteolysis; and group 885 represents a sel1 repeat family protein involved in signal transduction (17). RsxC is a component of a membrane-bound complex that couples electron transfer with the movement of ions across the membrane.

Most of the recombination events were shorter in length (<300 bp), and the larger recombination events (>1,000 bp) occurred less frequently (Fig. 4C), which indicates that recombination genes in *P. hangzhouensis* are more inclined to frequent replacement of short DNA fragments.

### Distribution of ESBL and carbapenem-resistance genes

**Fig 5:**
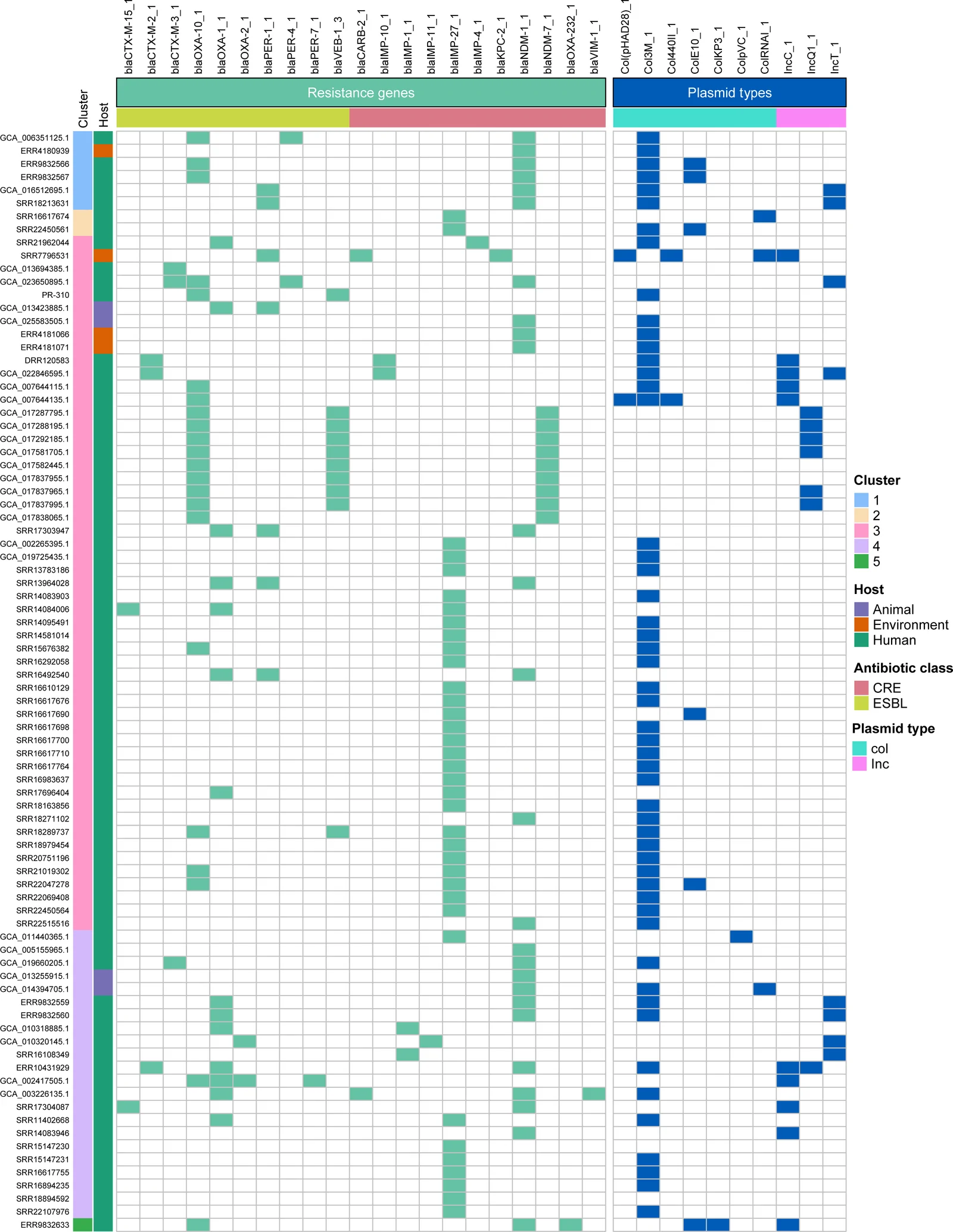
Distributions of ESBL and carbapenem-resistance genes, and plasmids’ replicon types in *P. hangzhouensis*. The presence of the trait category is represented by the colored squares.

The presence of extended-spectrum β-lactamase (ESBL) and carbapenem-resistance genes frequently results in beta-lactam drug resistance in clinic; therefore, we investigated the distribution of these genes in *P. hangzhouensis*. A total of 84 of 99 strains (84.85%) were detected to carry these genes, and 38 of them (45.24%; 38/84) carry both types (Fig. 5). More precisely, ESBL genes were detected in 44 isolates, with *bla*_OXA-10_ the most prevalent (*n* = 22), followed by *bla*_OXA-1_ (*n* = 14). Carbapenem-resistance genes were detected in 78 isolates, among which *bla*_IMP-27_ (*n* = 36) and *bla*_NDM-1_ (*n* = 26) were the most predominant. Moreover, we analyzed the distribution of plasmids in bacteria harboring these genes. The most prevalent plasmids belonged to the col3M group (*n* = 52), followed by, IncC type (*n* = 10), IncT plasmids (*n* = 8), and IncQ1 plasmids (*n* = 7).

### Genetic context of *bla*_IMP-27_

**Fig 6:**
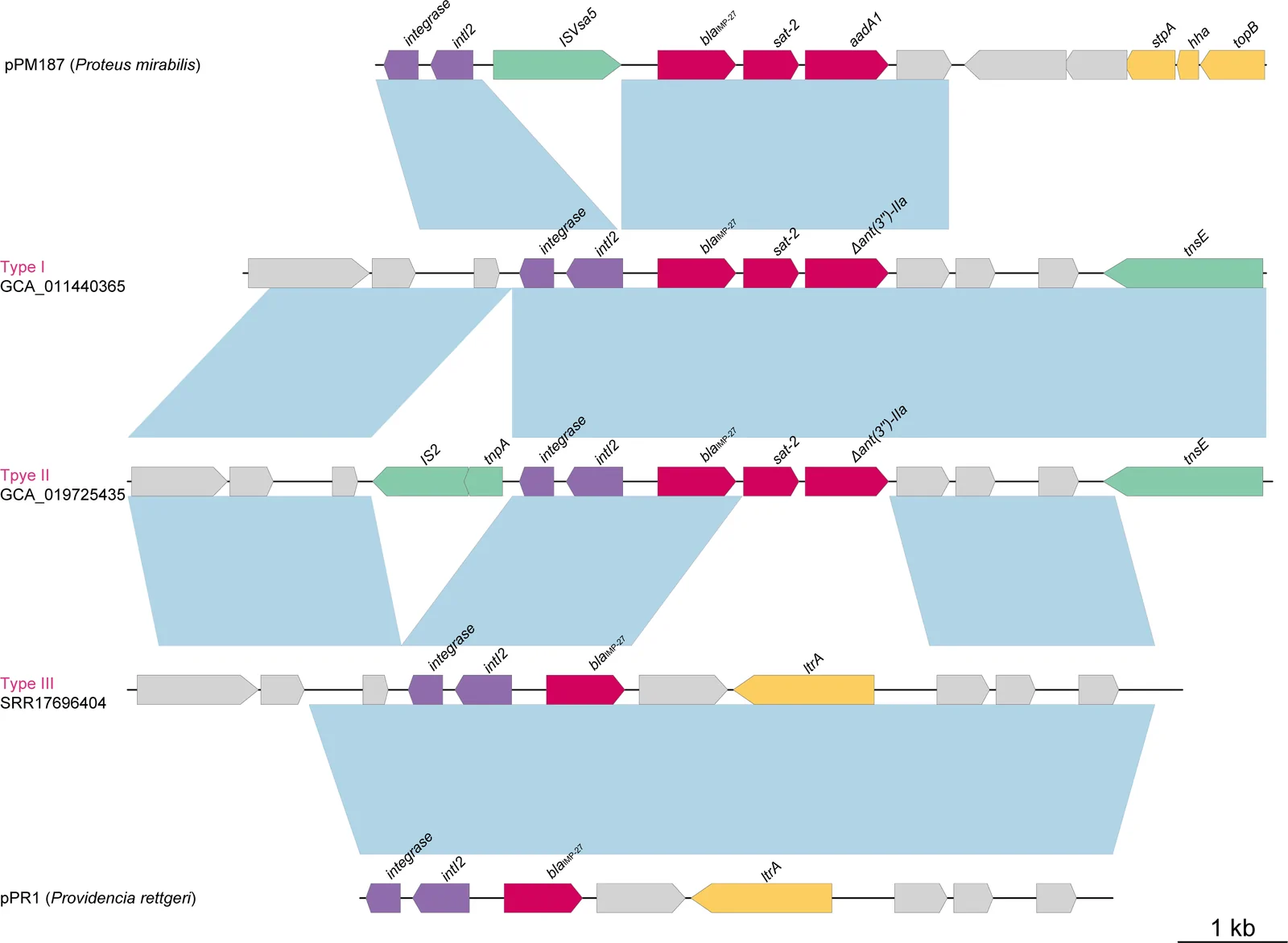
Schematic diagram of the genetic context surrounding *bla*_IMP-27_. Genes are illustrated as arrows and colored according to their function classification. The resistance genes are depicted in red, mobile elements are represented in shades of purple and green, other functional genes are illustrated in yellow, and genes without direct annotation are expressed in gray.

To obtain a better insight into the most prevalent carbapenem-resistance gene *bla*_IMP-27_, we analyzed all contigs (length > 10 kb) carrying this gene and observed three different types of genetic environments of *bla*_IMP-27_ (Fig. 6). In all these genetic contexts, *bla*_IMP-27_ was found to be located in an *Intl2* Integron. The contexts of type 1 and type 2 imipenem-hydrolyzing metallo-β-lactamase (IMP)-carrying sequences were similar to that of plasmid pPM187 (NOWA01000087.1) isolated from *Proteus mirabilis* (18), with the exception of an insertion sequence (IS2) fragment inserted downstream of the integrase in type 2. The composition of the downstream gene of *bla*_IMP-27_ in type 3 has changed considerably when compared to type 1 and type 2, which is consistent with the previously reported structure of pPR1 (NOWC01000095.1) from *P. rettgeri* (18).

## DISCUSSION

In this study, we first reported a novel species, *P. hangzhouensis*, characterized via comprehensive genome and phenotype analyses. The results from whole-genome ANI and dDDH analyses clearly indicated a significant divergence between *P. hangzhouensis* and other *Providencia* species with similarities falling well beneath the established species or subspecies threshold. Furthermore, differential biochemical properties effectively distinguished this species. The collective data strongly affirmed its standalone species status. Historically, *P. hangzhouensis* has been erroneously classified as *P. rettgeri* (19, 20), an error that has significantly obscured our understanding of its accurate epidemiology and clinical implications. Therefore, distinguishing *P. hangzhouensis* from *P. rettgeri* with precision, through phenotypic or genotypic methodologies, is of paramount importance. Currently, whole-genome-based ANI analysis is a reliable method for distinguishing different species (21), and the phylogeny based on the core genome also could provide a high resolution (Fig. 1B). Interestingly, our study identified two genes associated with metabolism specific to *P. hangzhouensis*, which hold potential for future rapid PCR identification and differentiation from *P. rettgeri*. Additionally, it is worth noting that a substantial number of genome sequences labeled as *P. rettgeri* in GenBank will need a correction to account for the newly identified species *P. hangzhouensis*.

Although *P. hangzhouensis* possessed a high degree of genetic relatedness with *P. rettgeri* and *P. huaxiensis* at the ANI level (91.54%–91.97%), it exhibited a higher global distribution and larger quantity. The phylogeny based on the core genome of the three species suggested that *P. hangzhouensis* might be the most recently diverged species (Fig. 1B). The timing of the sample records also indirectly supports this, which can be traced back to 2012 at the earliest (Table S3). Furthermore, it is important to note that *P. hangzhouensis* genomes had a higher proportion of clinical isolates (Fig. 1B). The minimum spanning tree (MST) based on the core SNPs (Fig. S3) indicated that this species has been widely spread within and between countries, highlighting the need for increased attention and surveillance.

The pan-genome analysis of *P. hangzhouensis* showed that its genome has a tendency to be open, indicating a high potential for the acquisition of new genes. COG functional classification analysis demonstrated a strong association between the pan-genomes of *P. hangzhouensis* and recombination. Additionally, the results obtained from fastGEAR indicated that recent recombination events were widespread in three species. Furthermore, a significant proportion (15.01%) of pan-genomes of *P. hangzhouensis* had a recombination history. These results suggest that the occurrence of recombination in *P. hangzhouensis* has contributed to the formation and diversity of this species.

It is worth noting that ESBL and carbapenem-resistance genes occurred at a high frequency in *P. hangzhouensis*, which could often lead to treatment failures in clinical settings. The most frequent carbapenem-resistance gene identified in this study was *bla*_IMP-27_, with a frequency of 46.15% (36/78). This gene was first reported in *Proteus mirabilis* in 2016 (22) and was subsequently found in *P. rettgeri* (18, 23), while Robert et al. (18) reported that the *bla*_IMP-27_ was located on transferable plasmids, which undoubtedly aggravated the risk of its transmission. Furthermore, other IMP-type metallo-β-lactamase-producing *P. hangzhouensis* isolates have also been reported in Japan (19). Given the high frequency of these carbapenem-resistance genes, it is crucial to pay close attention to their occurrence not only in nosocomial infections but also in other types of infections attributable to *P. hangzhouensis*.

Our study has limitations. Since nearly half of the isolates analyzed in this study were from the United States, and consequently, our findings may not fully represent the global molecular epidemiology of *P. hangzhouensis*. In addition, the strains we collected were primarily obtained from databases where the accuracy of collection time may be uncertain. We attempted to perform a Bayesian evolutionary analysis to estimate the date of origin for *P. hangzhouensis*, but unfortunately, no significant temporal signal was detected. Moreover, due to the previous misidentification of *P. hangzhouensis* as *P. rettgeri*, some uploaded plasmids that were labeled or characterized by the authors could not be accurately classified into the correct category, which posed great difficulties for us to conduct a detailed study of the plasmids.

In conclusion, our study identified a novel species, *P. hangzhouensis*, which was previously misidentified as *P. rettgeri*. We characterized the novel species through population genomics and analyzed its differences from *P. rettgeri* and *P. huaxiensis. P. hangzhouensis* frequently emerges with the emergence of ESBL and carbapenem-resistance genes, highlighting the importance of customized surveillance measures for this species in the future.

## MATERIALS AND METHODS

### Isolate collection and whole-genome sequencing

In August 2021, a strain of *Providencia rettgeri*, which is not commonly encountered in clinical settings but exhibited high resistance to cephalosporins, was isolated from a urine specimen of a patient. Genomic DNA of this isolate was extracted using an AxyPrep bacterial genomic DNA miniprep kit (Axygen Scientific, Union City, CA, USA). Whole-genome sequencing was performed on an Illumina HiSeq 2500 platform (paired-end run; 2 × 150 bp) and an Oxford Nanopore MinION platform. The publicly available genome assemblies and short-read data for *P. rettgeri* and *P. huaxiensis* were retrieved from the Assembly and SRA databases of NCBI using Kingfisher v7.6.1 (https://github.com/onevcat/Kingfisher) and ncbi-genome-download v0.3.1 (https://github.com/kblin/ncbi-genome-download), respectively. Assemblies with the label of poor by PATRIC (https://www.patricbrc.org/) and those derived from metagenomic isolates were excluded from the analysis.

### Genome assembly and species identification

Sequencing reads were subjected to adaptor trimming and quality filtering using fastp v0.23.2 (24). Trimmed reads were assembled using shovill v1.1.0 (https://github.com/tseemann/shovill) in the careful mode, and contigs with a length below 200 bp were filtered. The complete PR-310 genome was generated by synergizing the short-read data from Illumina sequencing and long-read data from Oxford Nanopore MinION sequencing through the Unicycler hybrid assembly pipeline (25). The quality of all assemblies was assessed using QUAST v5.2.0 (26), and assemblies with a contig count >300 were classified as low-quality genomes, and thus, excluded from subsequent analysis. The 16S rRNA gene sequences of PR-310 were procured through PCR, employing universal primers 27F and 1492R. Corresponding gene sequences from other species were acquired from the EzBioCloud database (12). These 16S rRNA gene sequences were compared using MAFFT v7.471 (27). Genomic ANIs were computed using OrthoANI (28) and *in silico* digital DNA-DNA hybridization index was assessed using genome-to-genome distance calculator (formula 2) (11). Table 1 enumerates the type strains of *Providencia* that are utilized for the purpose of species identification.

### Phenotypic characterization for the strain of novel species *Providencia hangzhouensis*

As previously described (29), the Gram-stain was performed and the biochemical characteristics of strain PR-310 were determined using the bioMérieux API 20E kits according to the manufacturer’s instructions. Oxidase activity was determined using an oxidase reagent (bioMérieux).

### Pan-genome analysis

Assemblies were annotated using Prokka v1.14.6 (30) with default settings. Pan-genome analysis for three species (*Providencia rettgeri*, *Providencia huaxiensis*, and *Providencia hangzhouensis*) was performed using Roary v3.13 (31) with a parameter of 100% coverage (i.e., only genes present in all isolates were thought to be core genes), respectively. The pan-genome’s decay parameter (*α*) was calculated using pagoo (32) and the accumulation curves of pan-genomes and core-genomes were visualized using ggplot2 (33). COG functional categories of the core, accessory, and unique gene sets of each species were functionally characterized using eggNOG-mapper v2.1.9 (34).

### Core-genome phylogeny

Core genes for all isolates of three species were determined by Roary with the parameters of a minimum of 90% blastp identity and 100% coverage. Nucleotide alignments of core genes were first generated by MAFFT v7.471 (27) and then concatenated to give a single core-genome alignment. TrimAl v1.4.1 (35) was used for further trimming and refinement of the alignment. Those clusters containing paralogous genes were filtered out of the final core gene alignment. Based on the refined core-genome alignment, an ML phylogeny tree was reconstructed using RaxML v8.2.12 (36), with a General Time Reversible (GTR) nucleotide substitution model selected as the optimal model by ModelTest-NG (37), and applying gamma correction with 1,000 bootstrap replications. The phylogenetic tree was visualized in ggtree (38) and phylogenetic network was detected using SplitsTree v4.14.5 (39) with NeighborNet algorithm base on the refined core-genome alignment. In order to search for genes specific to *P. hangzhouensis*, Roary was utilized to establish a minimum threshold value of 80% for the blastp threshold, as well as parameters that permitted paralogous division. Only those genes that were present in all isolates of *P. hangzhouensis* and absent in all isolates from other species were deemed to be unique genes (40).

### Recombination analysis

Genomic recombination events between three species were detected using fastGEAR (41) with default parameters. For further detecting the recombinations in *P. hangzhouensis*, fastGEAR was used to determine genes that undergo high-frequency recombination in the core genes and accessory genes. Recombined regions within the core genome were identified using Gubbins v3.3 (42) with the whole-genome alignment generated by Snippy v4.6.0 (https://github.com/tseemann/snippy) (CP029736.1 genome as a reference) as input file. A phylogenetic tree was constructed using RAxML based on the recombination-free core SNPs alignment with a GTR model and gamma correction (1,000 bootstrap replications). The population structure based on non-recombinant SNPs was estimated using RhierBAPS (43). The SNP-based MSTs were generated using PHYLOViZ v2.0 (https://online.phyloviz.net/index).

### Resistance genes and plasmid replicon type detection

Antimicrobial resistance genes and plasmid replicon types were identified using ABRicate v1.0.0 (https://github.com/tseemann/abricate) based on ResFinder, CARD, and PlasmidFinder database. Mobile genetic elements were identified using ISFinder (44) and INTEGRALL (45) with default parameters. ESBL and carbapenem-resistance genes were defined according to the BLDB database (46), and the presence or absence of these genes was visualized using the ComplexHeatmap (47). Comparisons of the nucleotide sequences were conducted using BLASTN and visualized in genoplotR (48).

## Data Availability

The information of strains used in this study is listed in Table S3. The complete genome sequences of PR-310 have been deposited in the GenBank database under BioProject accession no. PRJNA940527.

## SUPPLEMENTAL MATERIAL

Fig. S1-S4, Table of Contents of all Supplemental figures and tables spectrum.01227-23-s0001.docx Fig. S1-S4, Table of Contents of all Supplemental figures and tables.

Table S1-S3 spectrum.01227-23-s0002.xlsx Table S1-S3.

ASM does not own the copyrights to Supplemental Material that may be linked to, or accessed through, an article. The authors have granted ASM a non-exclusive, world-wide license to publish the Supplemental Material files. Please contact the corresponding author directly for reuse.

## References

[1] Wie S-H . 2015. Clinical significance of Providencia bacteremia or bacteriuria. Korean J Intern Med 30:167–169. doi:10.3904/kjim.2015.30.2.167 25750557 PMC4351322

[2] Koreishi AF , Schechter BA , Karp CL . 2006. Ocular infections caused by Providencia rettgeri. Ophthalmology 113:1463–1466. doi:10.1016/j.ophtha.2006.03.047 16797710

[3] Wang TKM , Ahn Y , Dunlop J . 2014. Providencia rettgeri peritonitis in a patient on peritoneal dialysis with perforated appendicitis. Perit Dial Int 34:569–570. doi:10.1177/089686081403400501 25075004 PMC4114679

[4] Sharma D , Sharma P , Soni P . 2017. First case report of Providencia rettgeri neonatal sepsis. BMC Res Notes 10:536. doi:10.1186/s13104-017-2866-4 29084590 PMC5663057

[5] Stackebrandt E , Goebel BM . 1994. Taxonomic note: a place for DNA-DNA reassociation and 16S rRNA sequence analysis in the present species definition in bacteriology. Int J Syst Evol Microbiol 44:846–849. doi:10.1099/00207713-44-4-846

[6] Mulet M , Lalucat J , García-Valdés E . 2010. DNA sequence-based analysis of the Pseudomonas species. Environ Microbiol 12:1513–1530. doi:10.1111/j.1462-2920.2010.02181.x 20192968

[7] Besser J , Carleton HA , Gerner-Smidt P , Lindsey RL , Trees E . 2018. Next-generation sequencing technologies and their application to the study and control of bacterial infections. Clin Microbiol Infect 24:335–341. doi:10.1016/j.cmi.2017.10.013 29074157 PMC5857210

[8] Ciufo S , Kannan S , Sharma S , Badretdin A , Clark K , Turner S , Brover S , Schoch CL , Kimchi A , DiCuccio M . 2018. Using average nucleotide identity to improve taxonomic assignments in prokaryotic genomes at the NCBI. Int J Syst Evol Microbiol 68:2386–2392. doi:10.1099/ijsem.0.002809 29792589 PMC6978984

[9] Goris J , Konstantinidis KT , Klappenbach JA , Coenye T , Vandamme P , Tiedje JM . 2007. DNA-DNA hybridization values and their relationship to whole-genome sequence similarities. Int J Syst Evol Microbiol 57:81–91. doi:10.1099/ijs.0.64483-0 17220447

[10] Richter M , Rosselló-Móra R . 2009. Shifting the genomic gold standard for the prokaryotic species definition. Proc Natl Acad Sci U S A 106:19126–19131. doi:10.1073/pnas.0906412106 19855009 PMC2776425

[11] Meier-Kolthoff JP , Auch AF , Klenk H-P , Göker M . 2013. Genome sequence-based species delimitation with confidence intervals and improved distance functions. BMC Bioinformatics 14:60. doi:10.1186/1471-2105-14-60 23432962 PMC3665452

[12] Yoon S-H , Ha S-M , Kwon S , Lim J , Kim Y , Seo H , Chun J . 2017. Introducing EzBioCloud: a taxonomically united database of 16S rRNA gene sequences and whole-genome assemblies. Int J Syst Evol Microbiol 67:1613–1617. doi:10.1099/ijsem.0.001755 28005526 PMC5563544

[13] Chun J , Oren A , Ventosa A , Christensen H , Arahal DR , da Costa MS , Rooney AP , Yi H , Xu X-W , De Meyer S , Trujillo ME . 2018. Proposed minimal standards for the use of genome data for the taxonomy of prokaryotes. Int J Syst Evol Microbiol 68:461–466. doi:10.1099/ijsem.0.002516 29292687

[14] Li Z , Liao F , Ding Z , Chen S , Li D . 2022. Providencia manganoxydans sp. nov., a Mn(II)-oxidizing bacterium isolated from heavy metal contaminated soils in Hunan Province, China. Int J Syst Evol Microbiol 72. doi:10.1099/ijsem.0.005474 35930465

[15] Medini D , Donati C , Tettelin H , Masignani V , Rappuoli R . 2005. The microbial pan-genome. Curr Opin Genet Dev 15:589–594. doi:10.1016/j.gde.2005.09.006 16185861

[16] Tettelin H , Riley D , Cattuto C , Medini D . 2008. Comparative genomics: the bacterial pan-genome. Curr Opin Microbiol 11:472–477. doi:10.1016/j.mib.2008.09.006 19086349

[17] Mittl PRE , Schneider-Brachert W . 2007. Sel1-like repeat proteins in signal transduction. Cell Signal 19:20–31. doi:10.1016/j.cellsig.2006.05.034 16870393

[18] Potter RF , Wallace MA , McMullen AR , Prusa J , Stallings CL , Burnham CAD , Dantas G . 2018. bla_IMP-27_ on transferable plasmids in Proteus mirabilis and Providencia rettgeri. Clin Microbiol Infect 24:1019. doi:10.1016/j.cmi.2018.02.018 PMC6105362 29496594

[19] Iwata S , Tada T , Hishinuma T , Tohya M , Oshiro S , Kuwahara-Arai K , Ogawa M , Shimojima M , Kirikae T . 2020. Emergence of carbapenem-resistant Providencia rettgeri and Providencia stuartii producing IMP-type metallo-β-lactamase in Japan. Antimicrob Agents Chemother 64:e00382-20. doi:10.1128/AAC.00382-20 32816727 PMC7577129

[20] Tchuinte PLS , Rabenandrasana MAN , Ramparany L , Ratsima E , Enouf V , Randrianirina F , Collard J-M . 2020. Genome-based insights into the resistomes and mobilomes of two Providencia rettgeri strains isolated from wound infections in Madagascar. J Glob Antimicrob Resist 20:178–182. doi:10.1016/j.jgar.2019.07.013 31325615

[21] Wu W , Feng Y , Zong Z . 2020. Precise species identification for Enterobacter: a genome sequence-based study with reporting of two novel species, Enterobacter quasiroggenkampii sp. nov. and Enterobacter quasimori sp. nov. mSystems 5:e00527-20. doi:10.1128/mSystems.00527-20 32753511 PMC7406230

[22] Dixon N , Fowler RC , Yoshizumi A , Horiyama T , Ishii Y , Harrison L , Geyer CN , Moland ES , Thomson K , Hanson ND . 2016. IMP-27, a unique metallo-β-lactamase identified in geographically distinct isolates of Proteus mirabilis. Antimicrob Agents Chemother 60:6418–6421. doi:10.1128/AAC.02945-15 27503648 PMC5038328

[23] Saavedra SY , Montilla-Escudero E , Wiesner M , González MN , Hidalgo AM , Ovalle MV , Chala MDS , Duarte C . 2022. First identification of the bla_IMP-27_ gene in a clinical isolate of Providencia rettgeri in Colombia. J Glob Antimicrob Resist 30:428–430. doi:10.1016/j.jgar.2022.05.005 35569756

[24] Chen S , Zhou Y , Chen Y , Gu J . 2018. Fastp: an ultra-fast all-in-one FASTQ preprocessor. Bioinformatics 34:i884–i890. doi:10.1093/bioinformatics/bty560 30423086 PMC6129281

[25] Wick RR , Judd LM , Gorrie CL , Holt KE . 2017. Unicycler: resolving bacterial genome assemblies from short and long sequencing reads. PLoS Comput Biol 13:e1005595. doi:10.1371/journal.pcbi.1005595 28594827 PMC5481147

[26] Gurevich A , Saveliev V , Vyahhi N , Tesler G . 2013. QUAST: quality assessment tool for genome assemblies. Bioinformatics 29:1072–1075. doi:10.1093/bioinformatics/btt086 23422339 PMC3624806

[27] Katoh K , Misawa K , Kuma K , Miyata T . 2002. MAFFT: a novel method for rapid multiple sequence alignment based on fast Fourier transform. Nucleic Acids Res 30:3059–3066. doi:10.1093/nar/gkf436 12136088 PMC135756

[28] Lee I , Ouk Kim Y , Park S-C , Chun J . 2016. OrthoANI: an improved algorithm and software for calculating average nucleotide identity. Int J Syst Evol Microbiol 66:1100–1103. doi:10.1099/ijsem.0.000760 26585518

[29] Kamlage B . 1996. Methods for general and molecular Bacteriology. edited by P. Gerhardt, R. G. E. Murray, W. A. wood and N. R. Krieg. 791 pages, numerous figures and tables. American society for Microbiology, Washington, D.C., 1994. price: 55.00 £, Vol. 40, p 103.

[30] Seemann T . 2014. Prokka: rapid prokaryotic genome annotation. Bioinformatics 30:2068–2069. doi:10.1093/bioinformatics/btu153 24642063

[31] Page AJ , Cummins CA , Hunt M , Wong VK , Reuter S , Holden MTG , Fookes M , Falush D , Keane JA , Parkhill J . 2015. Roary: rapid large-scale prokaryote pan genome analysis. Bioinformatics 31:3691–3693. doi:10.1093/bioinformatics/btv421 26198102 PMC4817141

[32] Ferrés I , Iraola G . 2021. An object-oriented framework for evolutionary pangenome analysis. Cell Rep Methods 1:100085. doi:10.1016/j.crmeth.2021.100085 35474671 PMC9017228

[33] H W . 2016. ggplot2: Elegant Graphics for data analysis. Springer-Verlag New York.

[34] Cantalapiedra CP , Hernández-Plaza A , Letunic I , Bork P , Huerta-Cepas J . 2021. eggNOG-mapper v2: functional annotation, orthology assignments, and domain prediction at the metagenomic scale. Mol Biol Evol 38:5825–5829. doi:10.1093/molbev/msab293 34597405 PMC8662613

[35] Capella-Gutiérrez S , Silla-Martínez JM , Gabaldón T . 2009. trimAl: a tool for automated alignment trimming in large-scale phylogenetic analyses. Bioinformatics 25:1972–1973. doi:10.1093/bioinformatics/btp348 19505945 PMC2712344

[36] Stamatakis A . 2014. RAxML version 8: a tool for phylogenetic analysis and post-analysis of large phylogenies. Bioinformatics 30:1312–1313. doi:10.1093/bioinformatics/btu033 24451623 PMC3998144

[37] Darriba D , Posada D , Kozlov AM , Stamatakis A , Morel B , Flouri T . 2020. ModelTest-NG: a new and scalable tool for the selection of DNA and protein evolutionary models. Mol Biol Evol 37:291–294. doi:10.1093/molbev/msz189 31432070 PMC6984357

[38] Yu G , Smith DK , Zhu H , Guan Y , Lam TT-Yuk . 2017. ggtree: an R package for visualization and annotation of phylogenetic trees with their covariates and other associated data. Methods Ecol Evol 8. doi:10.1111/2041-210X.12628

[39] Huson DH , Bryant D . 2006. Application of phylogenetic networks in evolutionary studies. Mol Biol Evol 23:254–267. doi:10.1093/molbev/msj030 16221896

[40] Luo T , Xu P , Zhang Y , Porter JL , Ghanem M , Liu Q , Jiang Y , Li J , Miao Q , Hu B , Howden BP , Fyfe JAM , Globan M , He W , He P , Wang Y , Liu H , Takiff HE , Zhao Y , Chen X , Pan Q , Behr MA , Stinear TP , Gao Q . 2021. Population genomics provides insights into the evolution and adaptation to humans of the waterborne pathogen Mycobacterium kansasii. Nat Commun 12:2491. doi:10.1038/s41467-021-22760-6 33941780 PMC8093194

[41] Mostowy R , Croucher NJ , Andam CP , Corander J , Hanage WP , Marttinen P . 2017. Efficient inference of recent and ancestral recombination within bacterial populations. Mol Biol Evol 34:1167–1182. doi:10.1093/molbev/msx066 28199698 PMC5400400

[42] Croucher NJ , Page AJ , Connor TR , Delaney AJ , Keane JA , Bentley SD , Parkhill J , Harris SR . 2015. Rapid phylogenetic analysis of large samples of recombinant bacterial whole genome sequences using Gubbins. Nucleic Acids Res 43:e15. doi:10.1093/nar/gku1196 25414349 PMC4330336

[43] Tonkin-Hill G , Lees JA , Bentley SD , Frost SDW , Corander J . 2018. RhierBAPS: an R implementation of the population clustering algorithm hierBAPS. Wellcome Open Res 3:93. doi:10.12688/wellcomeopenres.14694.1 30345380 PMC6178908

[44] Siguier P , Perochon J , Lestrade L , Mahillon J , Chandler M . 2006. ISfinder: the reference centre for bacterial insertion sequences. Nucleic Acids Res 34:D32–D36. doi:10.1093/nar/gkj014 16381877 PMC1347377

[45] Moura A , Soares M , Pereira C , Leitão N , Henriques I , Correia A . 2009. INTEGRALL: a database and search engine for integrons, integrases and gene cassettes. Bioinformatics 25:1096–1098. doi:10.1093/bioinformatics/btp105 19228805

[46] Naas T , Oueslati S , Bonnin RA , Dabos ML , Zavala A , Dortet L , Retailleau P , Iorga BI . 2017. Beta-lactamase database (BLDB) – structure and function. J Enzyme Inhib Med Chem 32:917–919. doi:10.1080/14756366.2017.1344235 28719998 PMC6445328

[47] Gu Z , Eils R , Schlesner M . 2016. Complex heatmaps reveal patterns and correlations in multidimensional genomic data. Bioinformatics 32:2847–2849. doi:10.1093/bioinformatics/btw313 27207943

[48] Guy L , Kultima JR , Andersson SGE . 2010. genoPlotR: comparative gene and genome visualization in R. Bioinformatics 26:2334–2335. doi:10.1093/bioinformatics/btq413 20624783 PMC2935412

