# Correction for Dong et al., "Whole-genome sequencing provides insights into a novel species: *Providencia hangzhouensis* associated with urinary tract infections”

**DOI:** 10.1128/spectrum.00905-24

**Published:** 2024-05-23

**Authors:** Xu Dong, Yuyun Yu, Jiaying Liu, Dan Cao, Yanghui Xiang, Kefan Bi, Xin Yuan, Shengchao Li, Tiantian Wu, Ying Zhang

## AUTHOR CORRECTION

Volume 11, no. 5, e01227-23, 2023, https://doi.org/10.1128/spectrum.01227-23. Following the guidelines of the International Code of Nomenclature of Prokaryotes (ICNP), a taxonomy section for *Providencia hangzhouensis* is required to comply with the necessary standards. Thus, the following taxonomic description should be inserted at the end of Results.

### Description of *Providencia hangzhouensis* sp. nov.

*Providencia hangzhouensis* (hang.zhou.en’sis. N.L. fem. adj. *hangzhouensis*, of Hangzhou, referring to Hangzhou City, Zhejiang Province, where the organism was isolated).

The cells are Gram stain-negative, motile, facultatively anaerobic rods, and negative for oxidase activity. Colonies appear circular, raised, yellow, opaque, and smooth after 24 h of incubation at 37°C on nutrient agar. Produces acid from D-glucose, D-mannitol, inositol, L-rhamnose, and amygdalin, but not from D-sorbitol, sucrose, melibiose, or D-arabinose. Tests positive for deaminase activity but negative for β-galactosidase, arginine dihydrolase, ornithine decarboxylase, lysine decarboxylase, and gelatinase. Additionally, it is positive for urease activity, indole production, and Voges-Proskauer reaction, and can utilize citrate, but does not produce H_2_S. *Providencia hangzhouensis* can be distinguished from all other *Providencia* species by its positive response to acetoin production and citrate utilization tests, while it is unable to metabolize melibiose.

The type strain, PR-310^T^ (= CCTCC AB 2023265^T^ = NBRC 116613^T^), was isolated from a patient with a urinary tract infection in Hangzhou, Zhejiang Province, China. The DNA G+C content of the type strain is 40.26%.

