# Supplementary material for: Whole-genome sequencing provides insights into a novel species: Providencia hangzhouensis associated with urinary tract infections
Source: Microbiol Spectr. 2023 Sep 21;11(5):e01227-23. doi: 10.1128/spectrum.01227-23 (PMC10581081; doi:10.1128/spectrum.01227-23)
Supplement: Fig. S1-S4, Table of Contents of all Supplemental figures and tables [file spectrum.01227-23-s0001.docx]

**Supplementary File 1: Figures**

**Table of Contents:**

**Fig. S1** Rooted maximum-likelihood trees representing the *Providencia* genus were constructed based on 16S rRNA gene sequences and whole-genome sequences.

**Fig. S2** A maximum-likelihood phylogenetic tree based on non-recombinant SNPs of *P. hangzhouensis.*

**Fig. S3** Minimum spanning trees of 99 *P. hangzhouensis* isolates.

**Fig. S4** Genomic mosaics arising from both ancestral and recent recombination events among the core genomes of the three species.

**Table S1**: Genetic information of four species-specific genes for *P. hangzhouensis*.

**Table S2**: Information about recombinant genes in *P. hangzhouensis*. The number represents the times that the gene has undergone recombination.

**Table S3**: Metadata for the isolates included in this study.

**Supplementary Figures:**

**
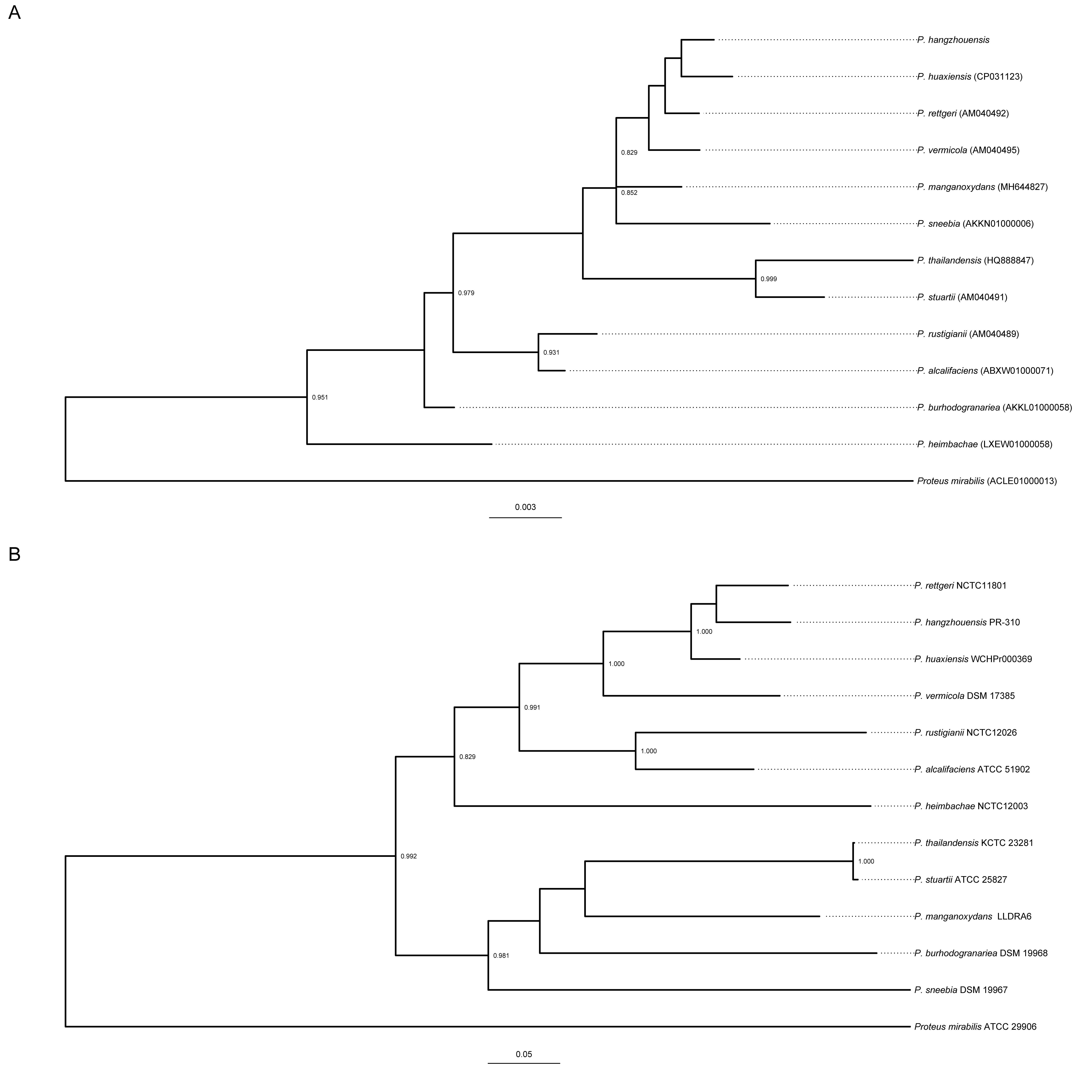
**

**Figure S1.** Rooted maximum-likelihood trees representing the *Providencia* genus were constructed based on 16S rRNA gene sequences (A) and whole-genome sequences (B). The sequence of *Proteus mirabilis* ATCC 29906T served as the outgroup. Bootstrap values, derived from 1,000 resamplings and >0.8, are denoted at the branch nodes.

**
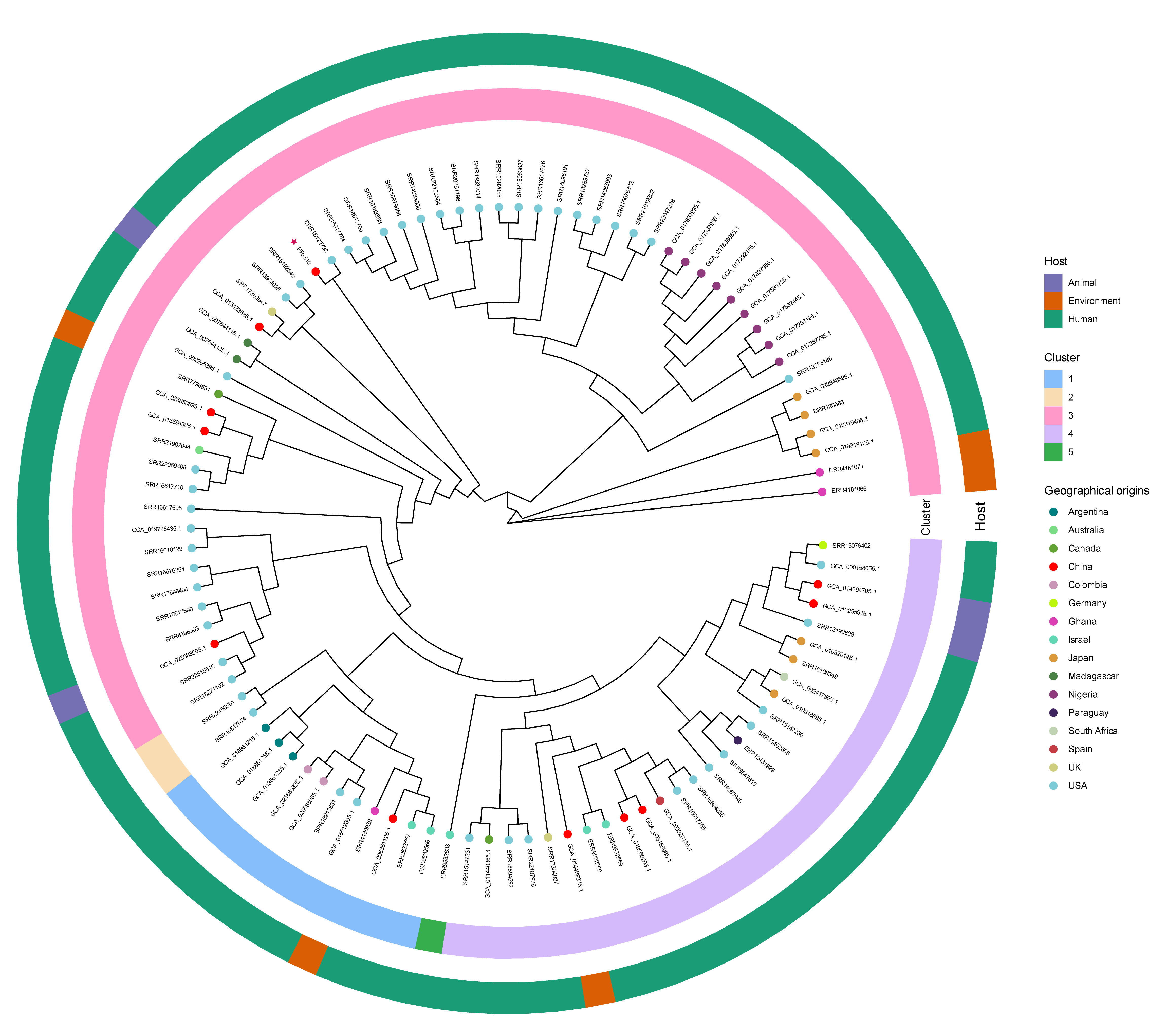
**

**Figure S2**. A maximum-likelihood phylogenetic tree based on non-recombinant SNPs of *P. hangzhouensis*. The colors of the terminal nodes correspond to the population structure estimated by RhierBAPS.


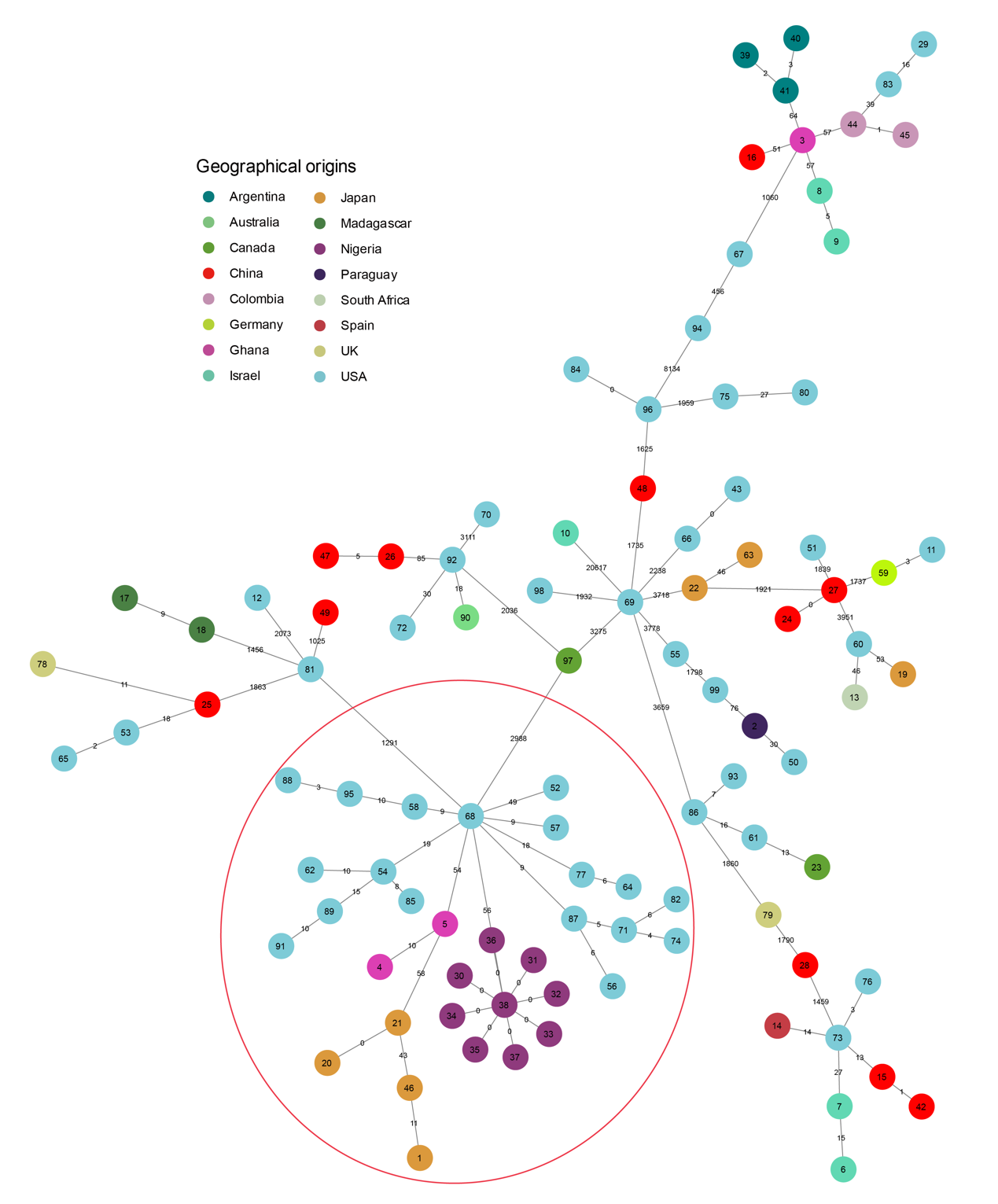


**Figure S3.** Minimum spanning trees of 99 *P. hangzhouensis* isolates. Each circle represents an isolate, with filled colors based on geographic origin. The connecting lines indicate the number of SNPs in a pairwise comparison. The major transmission cluster is circled in red.


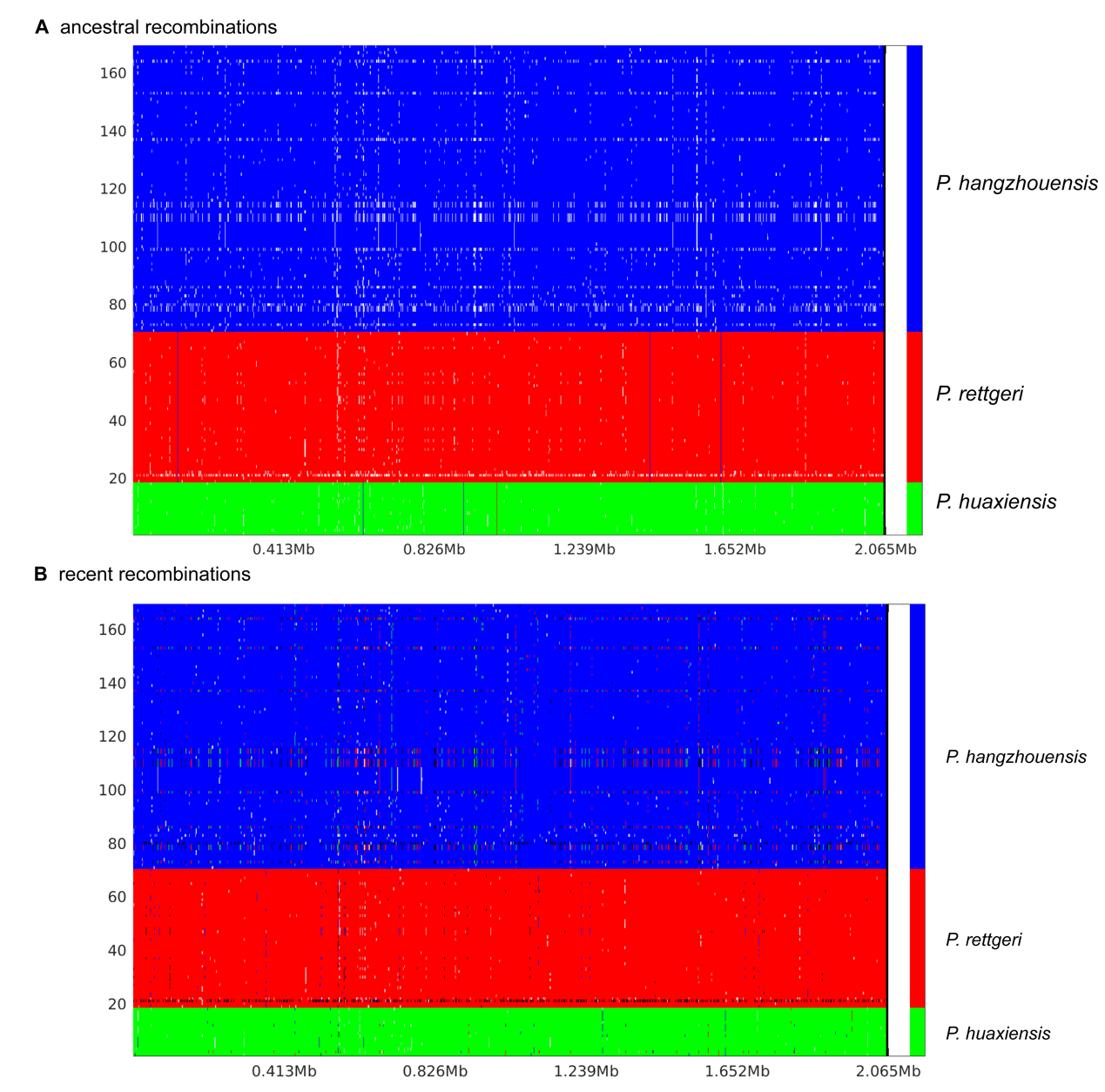


**Figure S4**. Genomic mosaics arising from both ancestral (A; upper panel) and recent (B; lower panel) recombination events among the core genomes of the three species. Each panel showcases a multiple alignment of 169 sequences. On the right side of each panel, three different species are represented by individual colors. The presence of differently colored fragments interspersed within the primary color of each species signifies past recombination events. Particularly in the panel illustrating ancestral recombination, the most recent recombinant fragments are denoted by white lines.

**Supplementary Tables: (Tables found in Supplementary File 2)**

**Table S1**: Genetic information of four species-specific genes for *P. hangzhouensis*.

**Table S2**: Information about recombinant genes in *P. hangzhouensis*. The number represents the times that the gene has undergone recombination.

**Table S3**: Metadata for the isolates included in this study.
